# Supplementary material for: HNF4A and HNF1A exhibit tissue specific target gene regulation in pancreatic beta cells and hepatocytes
Source: Nat Commun. 2024 Jun 22;15:4288. doi: 10.1038/s41467-024-48647-w (PMC11193738; doi:10.1038/s41467-024-48647-w)
Supplement: Supplementary file 1 — Supplementary Information [file 41467_2024_48647_MOESM1_ESM.pdf]

Supplementary Information

HNF4A and HNF1A exhibit tissue specific target gene regulation in pancreatic beta cells and hepatocytes

Ng, N.H.J. et al.

Supplementary Fig. 1

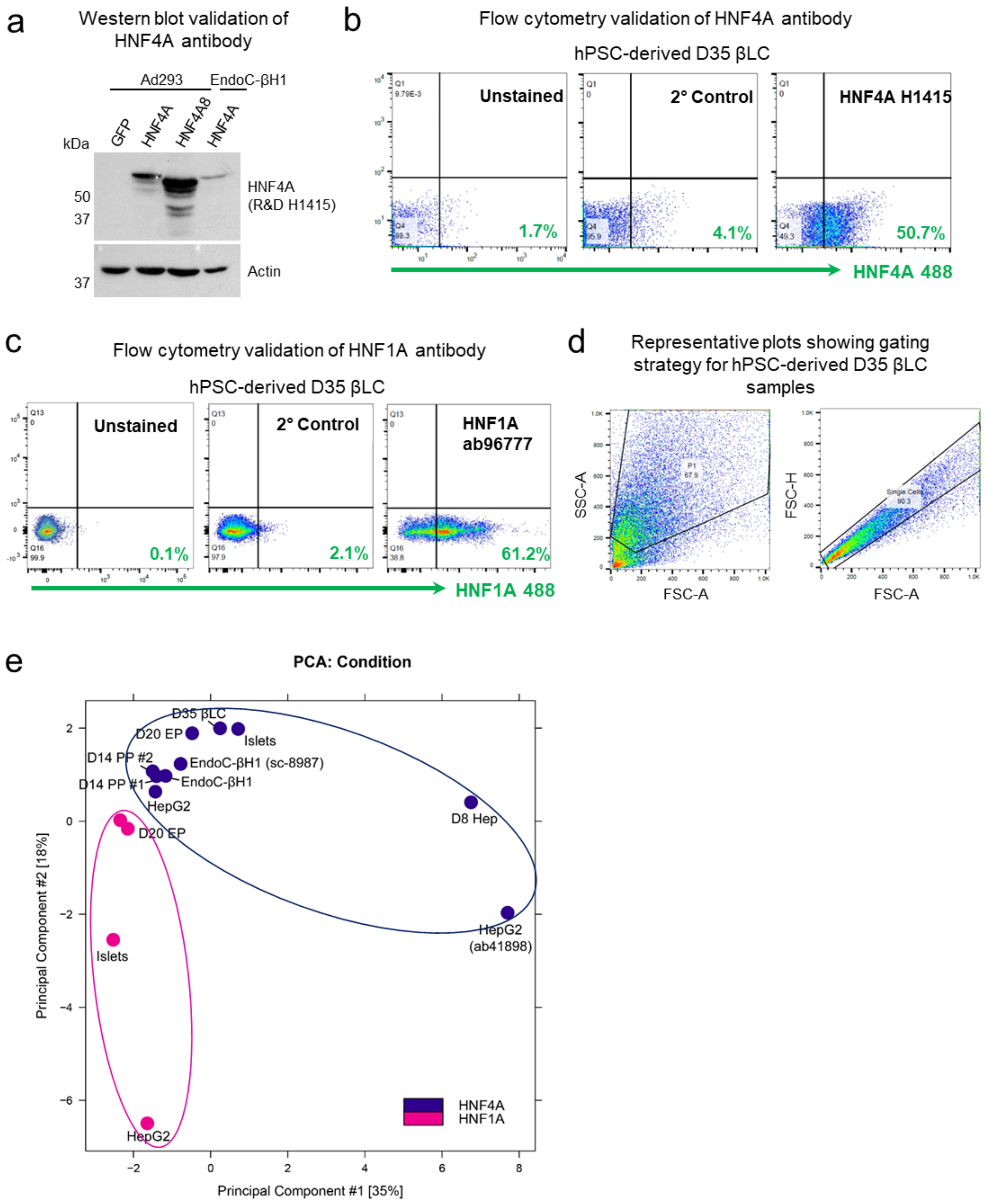

Supplementary Figure 1. Validation of the ChIP antibodies used and Principal Component Analysis (PCA) of the ChIP-Seq data.

a. Western blot analysis of HNF4A expression using the antibody for ChIP (R&D Systems, H1415) in Ad293 and EndoC-βH1 cells transiently overexpressing HNF4A or HNF4A8 WT.
b. Flow cytometry analysis of HNF4A expression using the antibody for ChIP (R&D Systems, H1415) in hPSC-derived D35 βLC.
c. Flow cytometry analysis of HNF1A expression using the antibody for ChIP (ab96777) in hPSC-derived D35 βLC.
d. Representative gating strategy for hPSC-derived D35 βLC for flow cytometry.
e. PCA plot of HNF4A and HNF1A ChIP-Seq samples included in this study.

Supplementary Fig. 2

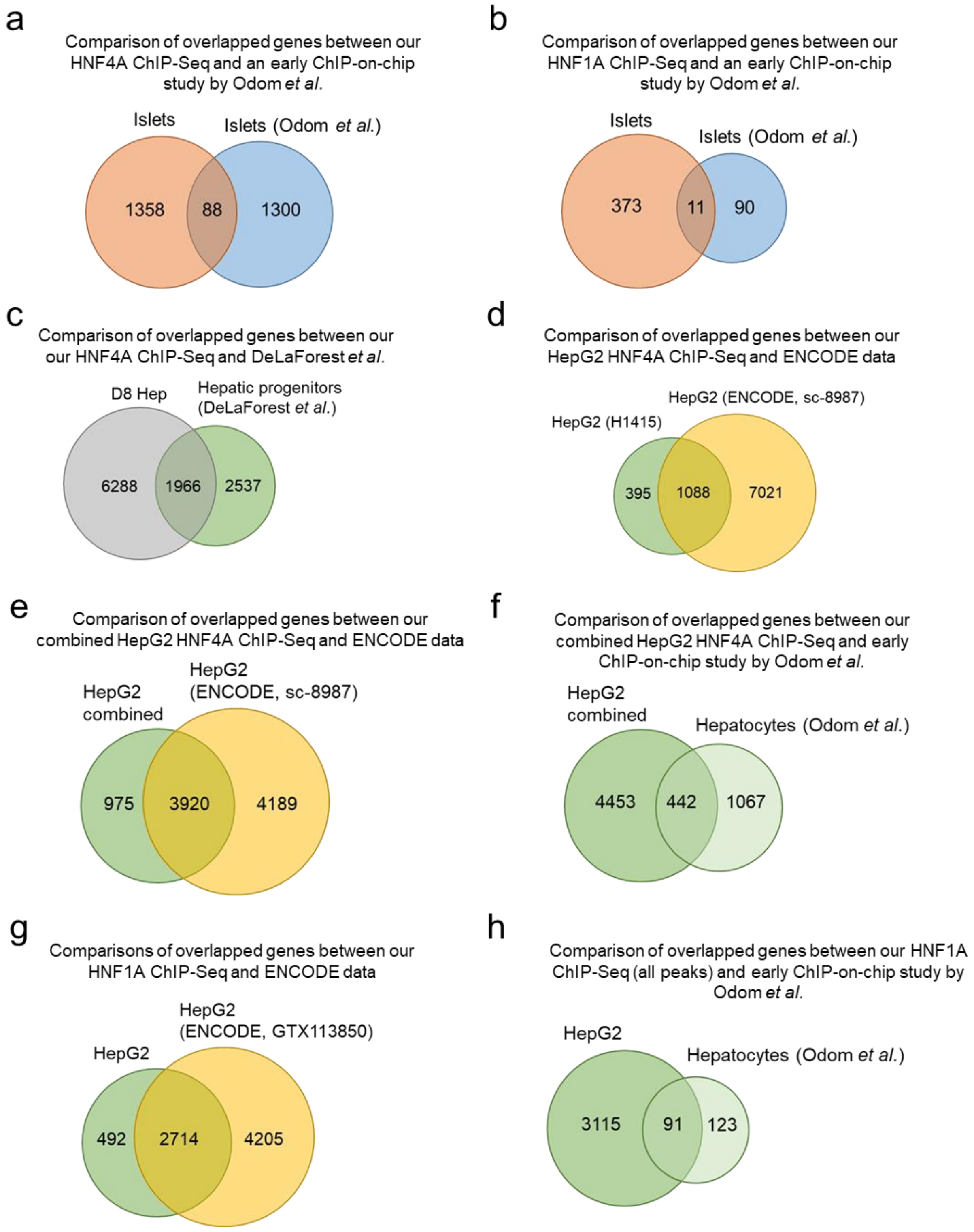

Supplementary Figure 2. Comparison between our ChIP-Seq datasets with existing published datasets for HNF4A and HNF1A ChIP.

a. Overlap between our HNF4A-bound targets in human islets and gene targets identified in the previously published ChIP-on-chip data by Odom et al. (2004).
b. Overlap between our HNF1A-bound targets in human islets and gene targets identified in the previously published ChIP-on-chip data by Odom et al. (2004).
c. Overlap between our HNF4A-bound targets in D8 Hep and gene targets identified in the previously published ChIP-Seq data by DeLaForest et al. (2018) (GSE104613).

d. Overlap between our HNF4A-bound targets in HepG2 (using R&D Systems H1415 antibody) and ENCODE data for HepG2 cells (ENCSR000BLF-ENCFF263RCV).
e. Overlap between a combined set of HNF4A-bound targets in HepG2 (expanded datasets from both H1415 and ab41898 antibodies) and ENCODE data for HepG2 cells (ENCSR000BLF-ENCFF263RCV).
f. Overlap between a combined set of HNF4A-bound targets in HepG2 (expanded datasets from H1415 and ab41898 antibodies) and those identified in a previously published ChIP-on-chip dataset by Odom et al. (2004) in primary hepatocytes.
g. Overlap between HNF1A-bound targets in HepG2 and ENCODE data for HepG2 cells (ENCSR800QIT-ENCFF162SGM).
h. Overlap between HNF1A-bound targets in HepG2 and those identified in a previously published ChIP-on-chip dataset by Odom et al. (2004) in primary hepatocytes.

Supplementary Fig. 3

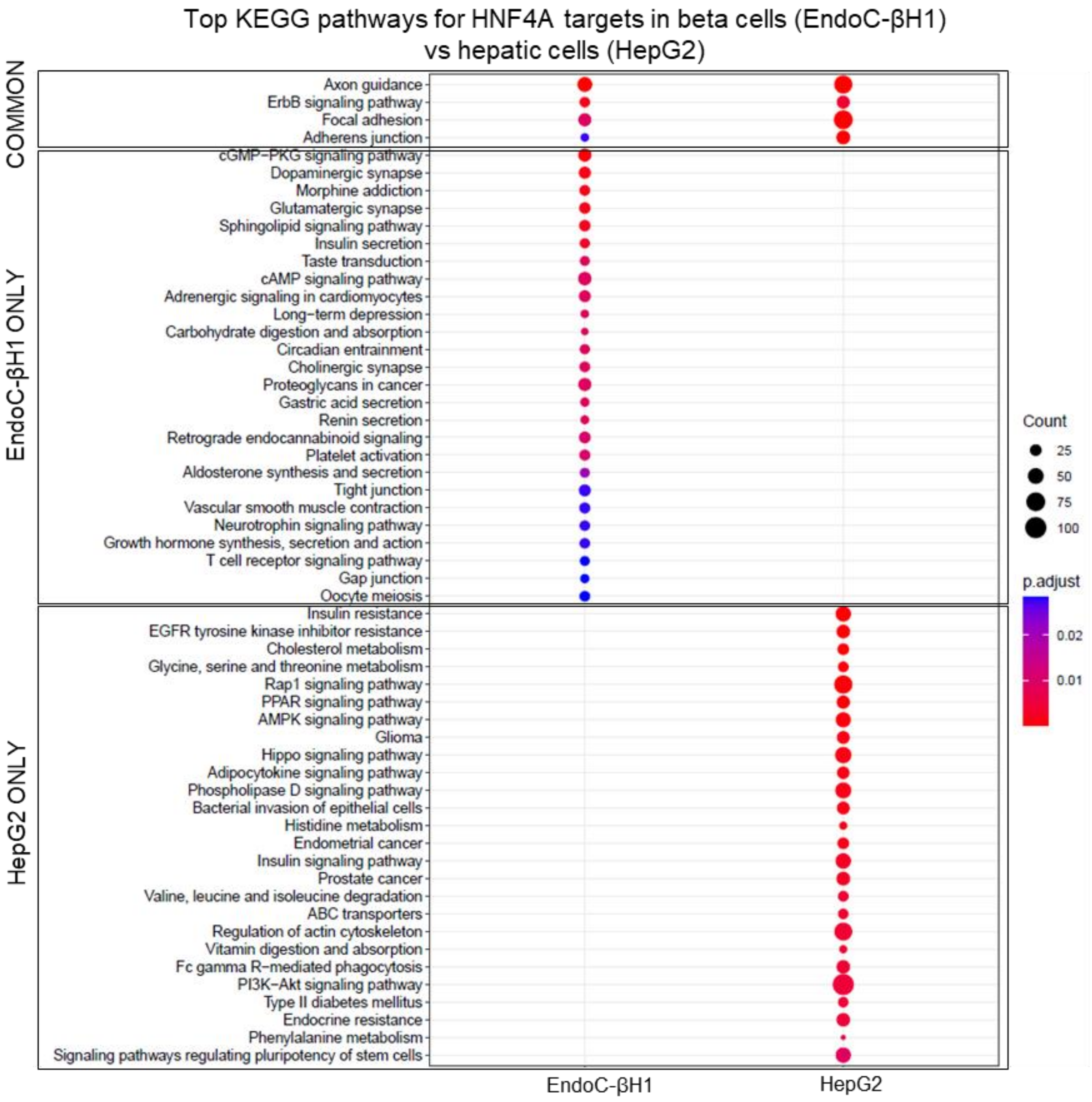

Supplementary Figure 3. Topmost common and distinct KEGG pathways enriched in HNF4A ChIP-Seq data in EndoC-βH1 and HepG2 cells. Analysis and visualization of pathway data is based on the ChIPseeker R package (see Methods).

Supplementary Fig. 4

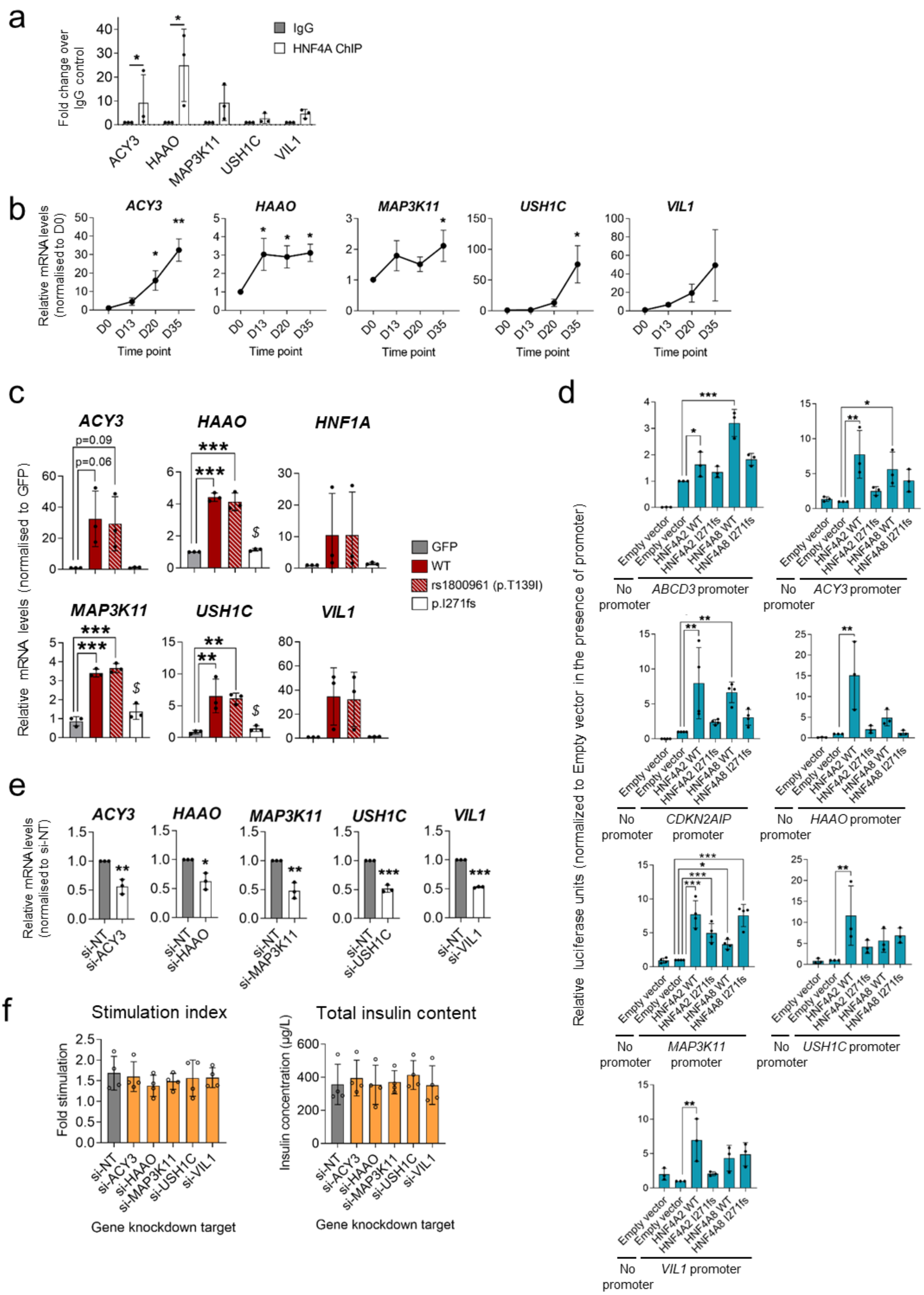

Supplementary Figure 4. Prioritization and functional validation of HNF4A-bound beta cell targets in Ad293 cells and human beta cells.

a. ChIP-qPCR validation at selected HNF4A target regions in beta cell samples (n=3). * indicates p<0.05 based on two-way ANOVA with Dunnett's multiple comparisons test.
b. Gene expression patterns of selected HNF4A target genes during beta cell differentiation from hPSCs over a 35-day time course (n=3 for *ACY3*/*USH1C*/*VIL1*; n=4 for *HAAO*/*MAP3K11*). * indicates p<0.05, ** indicates p<0.01 relative to D0, based on one-way ANOVA with Dunnett's post-hoc test.
c. Gene expression analyses of selected HNF4A target genes in Ad293 cells overexpressed with GFP (empty vector), HNF4A WT, T139I or MODY1 I271fs variant constructs (n=3). * indicates p<0.05, ** indicates p<0.01 relative to GFP; $ indicates p<0.01 relative to WT, using one-way ANOVA with Tukey's post-hoc test.
d. Transactivation activities at selected target promoters in Ad293 cells using luciferase reporter assays (n=3 for *ABCD3*/*ACY3*/*HAAO*/*USH1C*/*VIL1*; n=4 for *CDKN2AIP*/*MAP3K11*). * indicates p<0.05, ** indicates p<0.01, *** indicates p<0.001 relative to Empty vector in the presence of the promoter, using one-way ANOVA with Dunnett's multiple comparisons test.
e. Evaluation of the expression of selected target genes of interest upon siRNA-mediated knockdown of the respective target genes in EndoC-βH1 cells (n=3). * indicates p<0.05, ** indicates p<0.01, *** indicates p<0.001 relative to si-NT control, based on unpaired two-tailed Students' t test.
f. Stimulation index (insulin secretion at 16.7mM glucose normalized to insulin secretion at basal 2.8mM glucose) (left) and total insulin content (right) in EndoC-βH1 cells with siRNA-mediated knockdown of selected target genes (n=4).

Data are presented as mean ± SD. Each data point represents one independent experiment. Source data and exact *P* values are provided in the Source Data file.

Supplementary Fig. 5

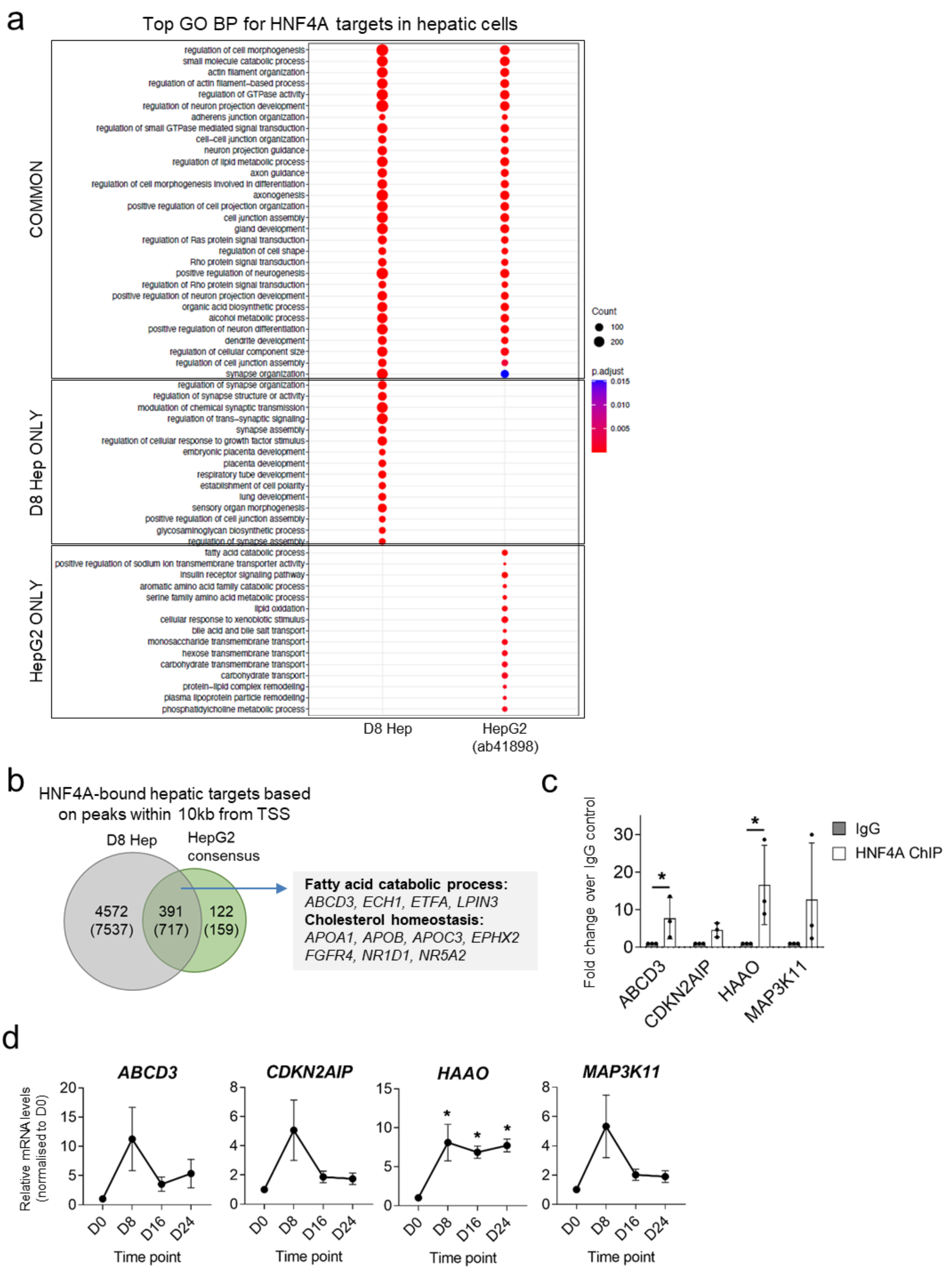

Supplementary Figure 5. HNF4A downstream targets signal transitions during hepatic development and pinpoint specific targets in hepatic cells for follow-up.

a. Topmost common and distinct GO BP for HNF4A ChIP-Seq targets in D8 hepatoblasts and HepG2 cells (using ab41898 antibody). Analysis and visualization of pathway data is based on the ChIPseeker R package (see Methods).
b. Venn diagram showing overlaps in HNF4A-bound target gene loci in D8 Hep and HepG2 datasets based on ChIP-Seq peaks within 10kb of the transcription start site (TSS) (all peaks in brackets).
c. HNF4A ChIP-qPCR fold enrichment at selected target promoters in hepatic cells (D8 Hep or HepG2) (n=3). * indicates $p<0.05$ based on two-way ANOVA with Dunnett's multiple comparisons test.
d. Gene expression patterns of selected HNF4A hepatic cell target genes during hepatocyte differentiation from hPSCs over a 24-day time course (n=4). * indicates $p<0.05$ relative to D0, based on one-way ANOVA with Dunnett's post-hoc test.

Data are presented as mean ± SD. Each data point represents one independent experiment. Source data and exact *P* values are provided in the Source Data file.

Supplementary Fig. 6

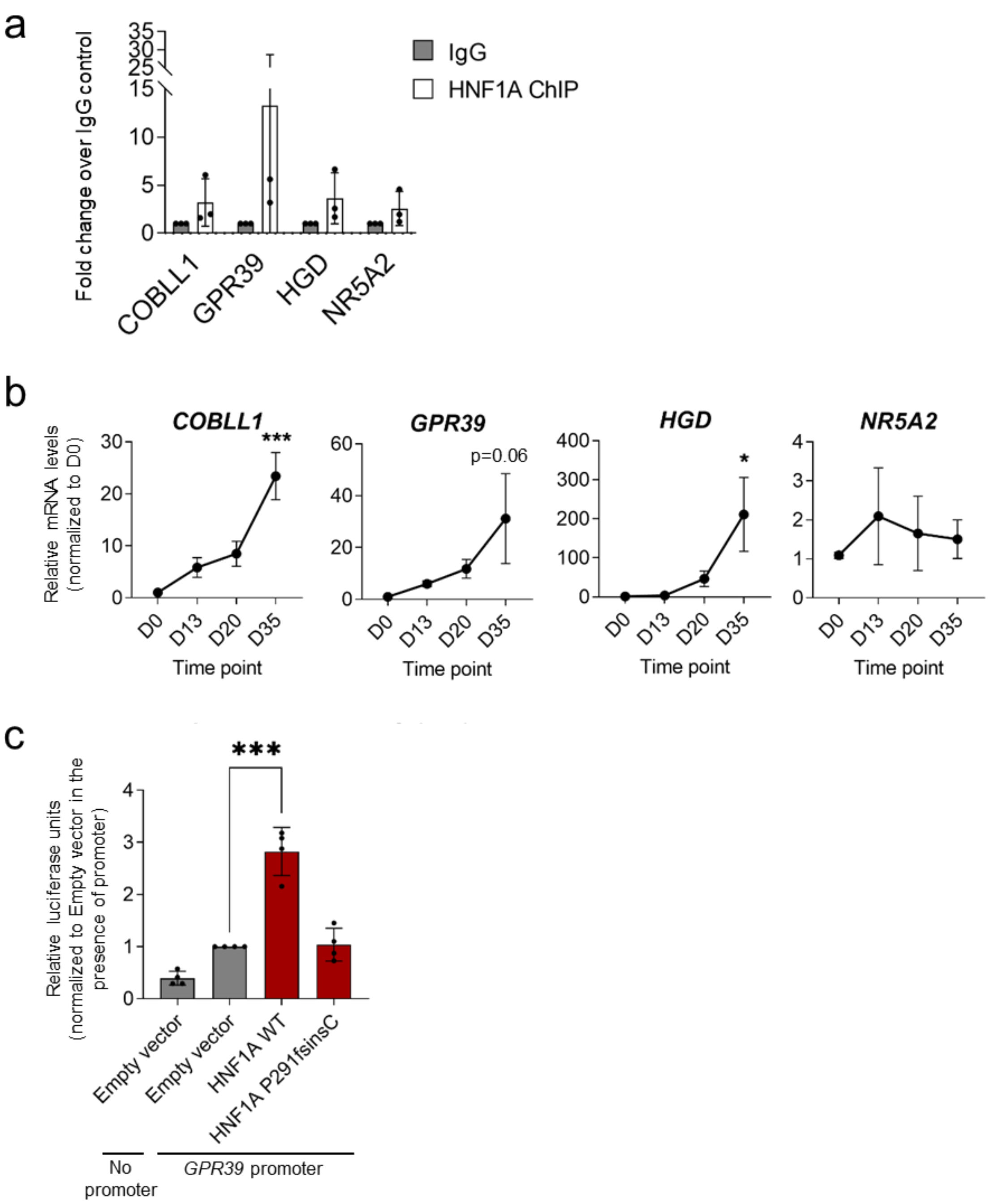

Supplementary Figure 6. Identification of HNF1A-bound targets in pancreatic endocrine cells highlight several target genes regulated by HNF1A.

a. HNF1A ChIP-qPCR fold enrichment at the *COBLL1*, *GPR39*, *HGD* and *NR5A2* promoters in human beta cell samples (n=3). * indicates p<0.05 based on two-way ANOVA with Dunnett's multiple comparisons test.
b. Gene expression patterns of selected HNF1A beta cell target genes during beta cell differentiation from hPSCs over a 35-day time course (n=4 for *COBLL1*/*HGD*/*NR5A2*; n=5 for *GPR39*). * indicates p<0.05, *** indicates p<0.001 relative to D0, based on one-way ANOVA with Dunnett's post-hoc test.
c. Transactivation analysis of *GPR39* promoter in Ad293 cells (n=4). *** indicates p<0.001 relative to Empty vector in the presence of the promoter, using one-way ANOVA with Tukey's post-hoc test.

Data are presented as mean ± SD. Each data point represents one independent experiment. Source data and exact *P* values are provided in the Source Data file.

Supplementary Fig. 7

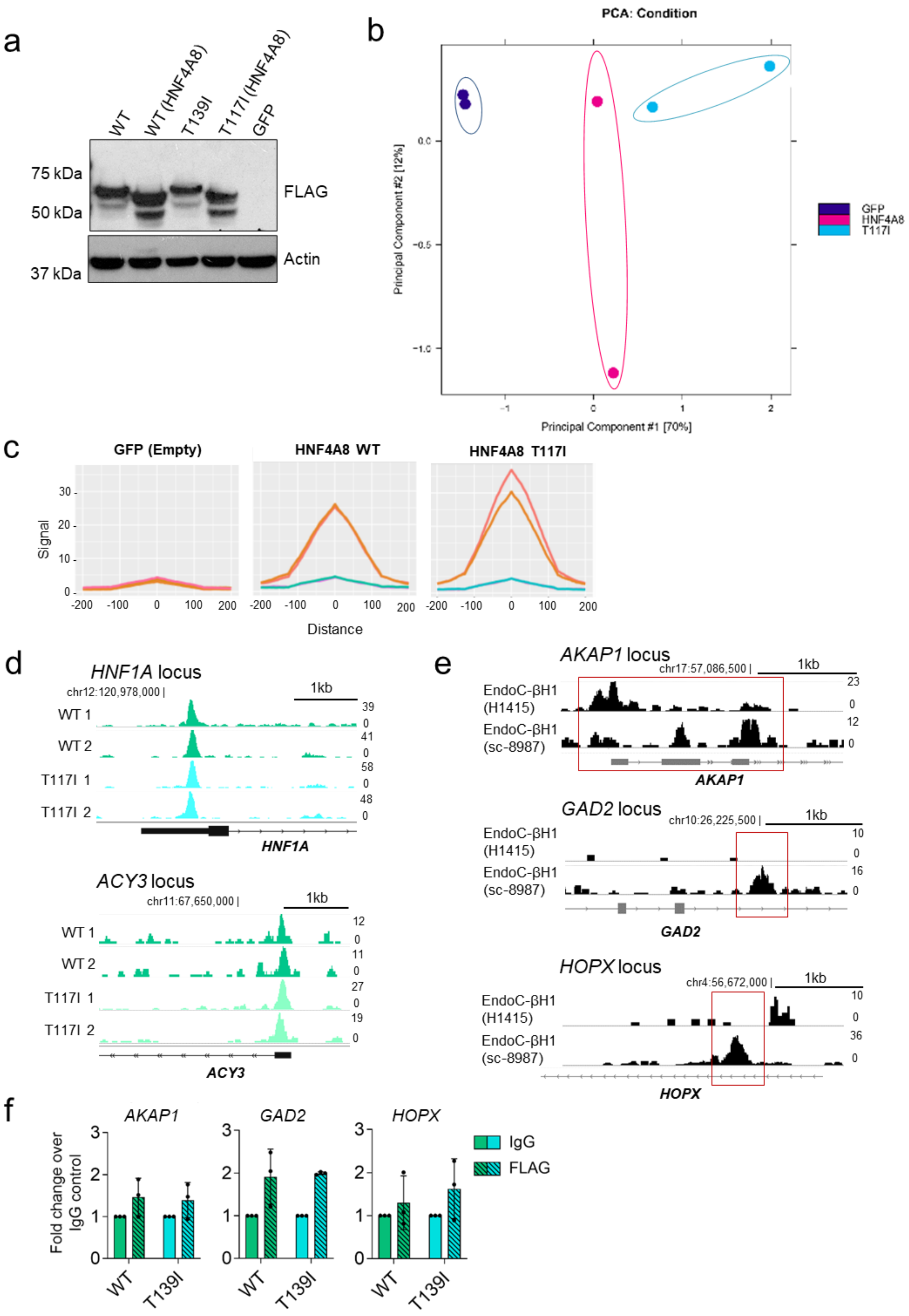

Supplementary Figure 7. Investigation of the effects of *HNF4A* T2D risk variant rs1800961 on gene regulation using FLAG ChIP-Seq.

a. Western blot analysis of cell lysates from Ad293 cells transiently overexpressing FLAG-tagged HNF4A WT and T2D variant constructs.
b. PCA plot of all FLAG ChIP-Seq samples from the EndoC-βH1 stable lines expressing GFP control (empty vector), HNF4A8 WT or HNF4A8 T117I in duplicate samples.
c. Signal detected within ChIP-Seq peaks in the EndoC-βH1 stable lines expressing GFP control (empty vector), HNF4A8 WT or HNF4A8 T117I.
d. IGV tracks showing FLAG ChIP-Seq peaks that map to the *HNF1A* and *ACY3* loci, in the EndoC-βH1 stable lines overexpressing WT or T117I. The scale used to visualize peaks in IGV is indicated on the right side of each track. The chromosomal location near the peak region is indicated.
e. IGV tracks showing HNF4A ChIP-Seq peaks that map to the *AKAP1*, *GAD2* and *HOPX* loci in the EndoC-βH1 samples. The scale used to visualize peaks in IGV is indicated on the right side of each track. The chromosomal location near the peak region is indicated.
f. FLAG ChIP-qPCR fold enrichment at the selected target regions in the stable EndoC-βH1 cell lines expressing HNF4A2 WT or T139I (n=3). Data are presented as mean ± SD. Each data point represents one independent experiment.

Source data and exact *P* values are provided in the Source Data file.

Supplementary Fig. 8 Uncropped Western Blot images

Uncropped Western Blot images for Supplementary Fig. 1a.

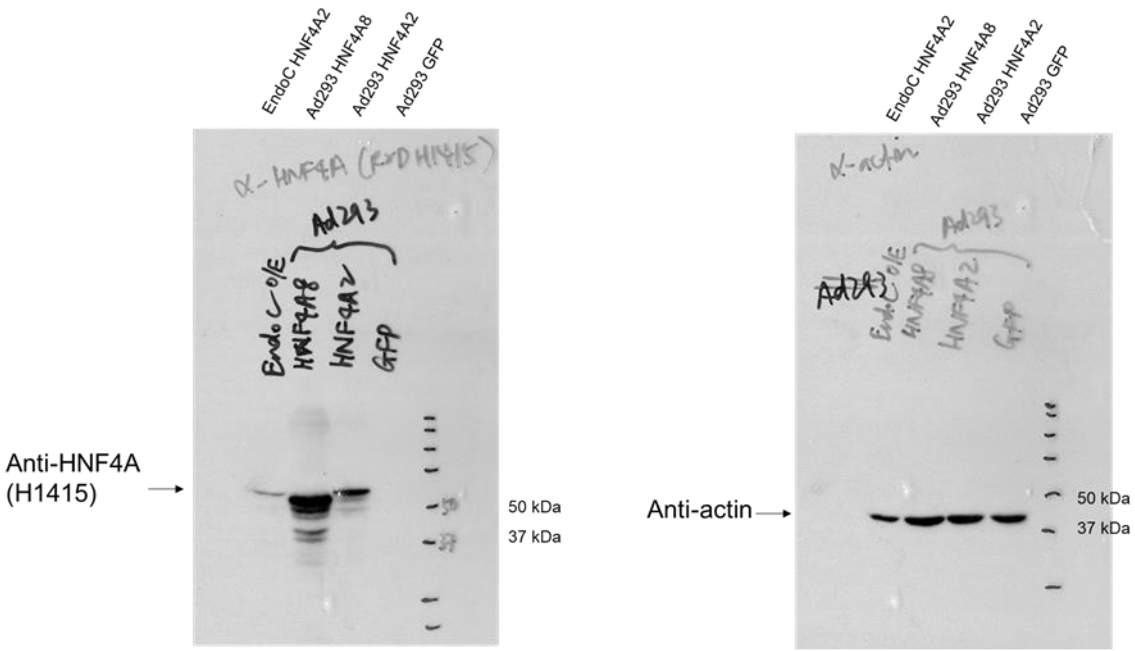

Uncropped Western Blot images for Supplementary Fig. 7a.

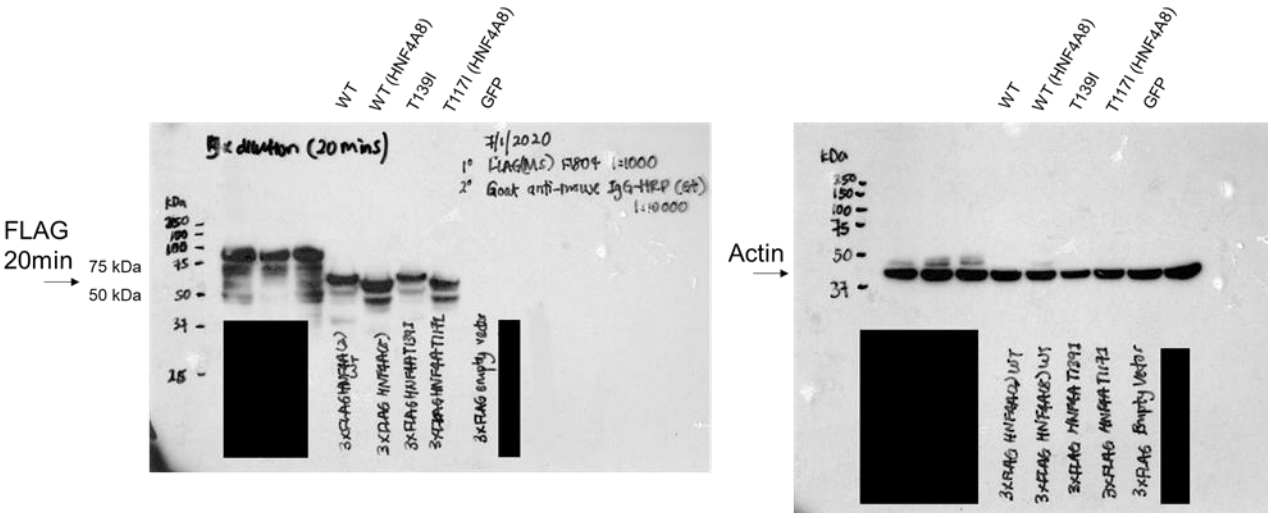

Supplementary Figure 8. Uncropped Western Blot images are provided as supporting information for Supplementary Figures 1a and 7a.

SUPPLEMENTARY DATA LEGENDS

Data S1. Peak enrichment regions identified from HNF4A ChIP-Seq across different cell types and in comparison with existing data. Related to Figures 1 and S2.

Data S2. Peak enrichment regions identified from HNF1A ChIP-Seq across different cell types and in comparison with existing data. Related to Figures 1 and S2.

Data S3. Gene ontology (GO) biological processes (BP) identified from HNF4A ChIP-Seq gene targets across multiple cell types. Analysis of GO data is based on the ChIPseeker R package (see Methods). Related to Figures 2, 6 and S3.

Data S4. Common HNF4A-bound beta cell gene targets identified from ChIP-Seq in D35 βLC, EndoC-βH1 cells and human islets (within 10kb upstream/downstream of TSS). Related to Figure 2.

Data S5. Common HNF4A-bound gene targets identified from ChIP-Seq in hepatic cells (D8 Hep and HepG2 cells). Related to Figures 4, 6 and S5.

Data S6. Gene ontology (GO) biological processes (BP) identified from HNF1A ChIP-Seq gene targets across multiple cell types. Analysis of GO data is based on the ChIPseeker R package (see Methods). Related to Figures 5 and 6.

Data S7. Common HNF1A-bound beta cell gene targets identified from ChIP-Seq in D20 EP and human islets (within 10kb upstream/downstream of TSS). Related to Figure 5.

Data S8. Common HNF4A- and HNF1A-bound gene targets identified from ChIP-Seq in human islets and HepG2 cells. Related to Figure 6.

Data S9. Components of the computed binding free energy (kcal mol−1) for HNF4A–DNA complexes. Related to Figure 7.

SUPPLEMENTARY TABLES

Table S1. Key Resources Table

REAGENT or RESOURCE	SOURCE	IDENTIFIER
Antibodies		
Mouse monoclonal anti-HNF4A	R&D Systems	H1415; RRID: AB_2263954
Mouse monoclonal anti-HNF4A	Abcam	ab41898; RRID: AB_732976
Rabbit polyclonal anti-HNF4A	Santa Cruz	sc-8987; RRID: AB_2116913
Rabbit polyclonal anti-HNF1A	Abcam	ab96777; RRID: AB_10679303
Mouse monoclonal anti-FLAG M2	Sigma Aldrich	F1804; RRID: AB_262044
Mouse monoclonal anti-β-Actin	Cell Signaling Technology	3700S; RRID: AB_2242334
Goat anti-rabbit IgG HRP	Promega	W4011; RRID: AB_430833
Goat anti-mouse IgG HRP	Santa Cruz	sc-2005; RRID: AB_631736
Donkey anti-goat IgG (H+L) Alexa Fluor 488	Thermo Fisher Scientific	A11055; RRID: AB_2534102
Donkey anti-mouse IgG (H+L) Alexa Fluor 488	Thermo Fisher Scientific	A21202; RRID: AB_141607
Donkey anti-rabbit IgG (H+L) Alexa Fluor 488	Thermo Fisher Scientific	A21206; RRID: AB_2535792
Donkey anti-mouse IgG (H+L) Alexa Fluor 594	Thermo Fisher Scientific	A21203; RRID: AB_141633
Bacterial and Virus Strains		
One Shot™ Stbl3™ Chemically Competent *E. coli*	Thermo Fisher Scientific	C737303
Biological Samples		
Human pancreatic islets	Alberta Islet Distribution Program, University of Alberta Hospital	H2144, H2148, H2154, H2191
Chemicals, Peptides, and Recombinant Proteins		
DMEM Low glucose with L-glutamine, sodium pyruvate	Hyclone	SH30021.01
DMEM High glucose with L-glutamine, sodium pyruvate	Hyclone	SH30243.01
DMEM Low glucose with pyruvate	Gibco	11885
MCDB131	Gibco	10372019
RPMI-1640	Gibco	11875093
mTeSR™1	STEMCELL Technologies	85850
TeSR™-E8™	STEMCELL Technologies	05990
CMRL-1066 supplemented	Mediatech	99-663-CV
KnockOut serum replacement (KOSR)	Thermo Fisher Scientific	10828-028
Fetal bovine serum (FBS)	Hyclone	SH30071.03
MEM non-essential amino acids (NEAA)	Thermo Fisher Scientific	11140-050
GlutaMAX Supplement	Thermo Fisher Scientific	350500061
Fibronectin	Sigma Aldrich	F1141
ECM Gel	Sigma Aldrich	E1270
Beta-mercaptoethanol (1000x)	Gibco	21985023
Miami medium	Corning	98-021-CV
Bovine serum albumin (BSA) fatty acid-free	Proliant	7500804
Bovine serum albumin (BSA)	Sigma Aldrich	A9418
TrypLE Express	Thermo Fisher Scientific	12604021
Collagenase IV	Thermo Fisher Scientific	17104-019
Dispase	STEMCELL Technologies	7923

Y-27632	STEMCELL Technologies	72304
B-27 without vitamin A	Thermo Fisher Scientific	12587-010
Oncostatin M	Miltenyi Biotec	130093976
HGF	Miltenyi Biotec	130093872
FGF2	Miltenyi Biotec	130-093-838
FGF7	Miltenyi Biotec	130037178
FGF10	Miltenyi Biotec	130093850
Activin A	R&D Systems	338-AC-050
CHIR 99021	Tocris	4423
LY294002	Tocris	L-7962
Retinoic Acid	Wako	18601114
Nicotinamide	Sigma Aldrich	N0636
BMP4	Miltenyi Biotec	130111168
Sant-1	Santa Cruz	sc-203253
Phorbol 12,13-dibutyrate (PdBU)	Tocris	4153
LDN193189	Sigma Aldrich	SML0559
XXI (Gamma-Secretase Inhibitor)	Merck	565790
Alk5 inhibitor II	Enzo	ALX-270-445
L-3,3’,5-triiodothyronine	Merck Millipore	642511
Betacellulin	Cell Signaling	5235
Insulin-Transferrin-Selenium-Ethanolamine (ITS-X)	Thermo Fisher Scientific	51500056
L-Ascorbic acid	Sigma Aldrich	A8960
Penicillin/Streptomycin (Pen/Strep)	Gibco	15140122
Heparin	Sigma Aldrich	H3149
Transferrin	Sigma Aldrich	T8158
Sodium Selenite	Sigma Aldrich	214485
Dimethyl 3,3'-dithiobispropionimidate	Sigma Aldrich	D2388
3,3’-Dithiodipropionic acid di(N-hydroxysuccinimide ester)	Sigma Aldrich	D3669
Chloroform 99.8+%	Thermo Fisher Scientific	10293850
4% Paraformaldehyde Phosphate Buffer Solution	Wako	161-20141
Leupeptin hemisulfate	Abcam	ab141404
PMSF	Thermo Fisher Scientific	36978
DTT (dithiothreitol)	Thermo Fisher Scientific	R0861
Glycine	1st Base	BIO-2085
Proteinase K	Thermo Fisher Scientific	EO0491
Phenol/Chloroform/Isoamyl Alcohol, 25:24:1	Merck Millipore	516726
Phusion High-Fidelity DNA Polymerase	Thermo Fisher Scientific	F530S
TRIzol™ Reagent	Thermo Fisher Scientific	15596018
Critical Commercial Assays		
iTaq Universal SYBR Green Supermix	Bio-Rad	172-5120
NucleoSpin RNA Kit	MN	740955
NucleoBond Xtra Midi kit	MN	740410-50
EZ-10 Spin Column Plasmid DNA Miniprep Kit	BioBasic	BS614
High-Capacity cDNA Reverse Transcription Kit	Applied Biosystems	4368813
Dual-Luciferase® Reporter Assay System	Promega	E1910
Insulin ELISA Kit	Mercodia	10-1113-10
Deposited Data		
ChIP-Seq data	This study	GEO: GSE206240
Experimental Models: Cell Lines		
Human: H9/WA09 hESC line (NIH approval number NIHhESC-10-0062)	WiCell	WAe0009-A

Human: iAGb	(Loo et al., 2020)	N/A
Human: Ad293	Agilent	STR-240085
Human: HepG2	ATCC	HB-8065
Human: EndoC-βH1	Univercell Biosolutions	N/A
Recombinant DNA		
Plasmid: pCDH-HNF4A2	(Ng et al., 2019)	N/A
Plasmid: pCDH-HNF4A2 I271fs	(Ng et al., 2019)	N/A
Plasmid: pCDH-HNF4A2 T139I	This study	N/A
Plasmid: pCDH-HNF4A8	(Ng et al., 2019)	N/A
Plasmid: pCDH-HNF4A8 I271fs	(Ng et al., 2019)	N/A
Plasmid: pCDH-HNF4A8 T117I	This study	N/A
Plasmid: pCDH-3xFLAG-Empty	This study	N/A
Plasmid: pCDH-3xFLAG HNF4A8	This study	N/A
Plasmid: pCDH-3xFLAG HNF4A8 T117I	This study	N/A
Plasmid: pcDNA3.1-HNF4A2	This study	N/A
Plasmid: pcDNA3.1-HNF4A2 I271fs	This study	N/A
Plasmid: pcDNA3.1-HNF4A2 T139I	This study	N/A
Plasmid: pcDNA3.1-HNF4A8	This study	N/A
Plasmid: pcDNA3.1-HNF4A8 I271fs	This study	N/A
Plasmid: pcDNA3.1-HNF4A8 T117I	This study	N/A
Plasmid: pCDH-HNF1A	(Low et al., 2021)	N/A
Plasmid: pCDH-HNF1A P291fsinsC	(Low et al., 2021)	N/A
Plasmid: pcDNA3.1-HNF1A	This study	N/A
Plasmid: pcDNA3.1-HNF1A P291fsinsC	This study	N/A
Sequence-Based Reagents		
Primers for qPCR, see Table S9	This paper	N/A
Primers for ChIP qPCR, see Table S10	This paper	N/A
Software and Algorithms		
FlowJo v7	N/A	https://www.flowjo.com/solutions/flowjo
BioRad CFX Manager Software v2.3	Bio-Rad	N/A
GraphPad Prism 9	GraphPad	http://www.graphpad.com/scientificsoftware/prism/
Integrative Genomics Viewer (IGV) v2.13.1	IGV	https://igv.org/
Other		
Olympus FV1000 inverted confocal microscope	Olympus	N/A
BD LSR II Flow Cytometer	BD Biosciences	N/A
CFX384 Touch™ Real-Time PCR Detection System	Bio-Rad	N/A

Table S2. Quantitative real-time PCR primers used in this study for ChIP validation.

**Gene/Locus**	**Forward Primer (5' to 3')**	**Reverse Primer (5' to 3')**
AKAP1	AACCCGGTGGACTTCGCTCC	ACGCGCACTACAGTAGGGGA
ABCD3	CGCGTTTCCGGAGACCCTGA	CAGCCTCCGTGGGCGGAAC
ACY3	CCTACCCCCGAAACAGCGGA	GAGGTGCCCTGCGCTGAAGTC
CDKN2AIP	CCGCTCACACACAGGCGTCTC	GCGGCGGCCAAAGTCCG
COBLL1	TGGTTTTCAACCTGACCTTGCCACT	AGAGTCACTGATAAGTCTGCTTGCC
GAD2	AGTGGCAGGAGCGGATGTCATT	TCCCCGCCTCATCATTTGCTTG
GAPDH	AATGTCACCGGGAGGATTGG	CCCCCAGCTACAGAAAGGTC
GPR39	TGCTGAGTCAAACCCCGCCG	CCAGCTGCCGGCACTTGAGA
HAAO	GGCGGGCTCAGACCAAAGTCC	AATCCCAAGCTCACGAGGCGG
HGD	GGGCATTACAGTGCCTTCGCTT	CTCCACCAGTGCCTGCCAGAAA
HNF1A	CCATAGCTCCCTGTCCCTCT	GCCCCTGCCTGTTCTGTTTA
HNF4A	ACTTGGGGTGACAATGGCTTGGA	ACAACTGCTGGGGCCCTAACTC
HOPX	AAGTCCAACCACCCATGCTGCT	GAGCTGGCTAAAGCTGCACACC
MAP3K11	CCCCCGGGGCCAAAGTACAAA	TGGGCCGAGACTGCCTGTTC
NR5A2	ACAGGGTCCTCTTATCAACCTGCAT	ACAAGCAAGTCTTTCCCTGGACTCT
USH1C	ACCTGGGAGGGGAAGGCTGT	CCTGTGTGCAGGCAGGGCAG
VIL1	GCCAGCTTGCCACAATTCCCTG	CCGTCCCCTCGCACCACCTA

Table S3. Quantitative real-time PCR primers used in this study for gene expression analysis.

Gene	Accession Number	Forward Primer (5' to 3')	Reverse Primer (5' to 3')
ACTIN	NM_001101.3	TTGCCGATCCGCCGCCCGTC	CCCATGCCCACCATCACGCCCTGG
ABCD3	NM_002858.4	CGGCCTGCACGGTAAGAAAAGTG	TGTCCACCACAGCTCGCTCC
ACY3	NM_080658.2	CGAACTCTTCAACCAGGGTACGGC	GGAGCACCAGGCTGCAGTGG
AKAP1	NM_003488.4, NM_001242902.1, NM_001370423.1	CTGCCGATGACATCCTGGCTCAT	AACCACTTGGGCTCGCCACC
CDKN2AIP	NM_017632.4	GCCTCCGCTAGCACGGATGA	GAGGGTATCGGCACCCGAGG
COBLL1	NM_001365672.2	ATCGCTTCAAGAGCTTGCCCCT	AGGGGCACTTGCAGTTTGGTCT
EEF1A1	NM_001402.6	TCCTGGCAAGCCCATGTGTGT	TGGTGACCTTGCCAGCTCCAG
GAD2	NM_000818.3	GCGTGGAGAGGGCCAACTCTG	CCCGGTAGTCCCCTTTGCCC
GPR39	NM_001508.3	GCAGACCATCATCTTCCTGAGGCT	GTCGTGCTTGGGTTTGGCCG
HAAO	NM_012205.3	GCAACAAGCTCATGCACCAGG	TGCCGAATGACCACATCCCG
HGD	NM_000187.3	GTGGCCTTTGACCATGCAGACC	TGGCAGGAACCCACCTTGCT
HNF1A	NM_001306179.1	CTTCTGCAGGAGGACCCGTGGCGT	GGCGGCCCGCTTCTGCGTCT
HNF4A	NM_000457.4, NM_175914.4	GGACGACCAGGTGGCCCTGCTCAGA	GCTCCGGGCAGTGCCGAGGGA
HOPX	NM_032495.6, NM_139211.5, NM_001145460.2	CAGGGACCATGTCGGCGGAG	AGCGTGGTGGAATCCGGGTG
MAP3K11	NM_002419.4	ATGGGAGGAGAAGGTCCCGCAT	TCCTCGGGCTCCAGGCTAGG
NR5A2	NM_001276464.2 NM_003822.5 NM_205860.3	TCACCTGTGAAAGCTGCAAGGGAT	TCCTCCACGCATTCGGTCGG
PAX4	NM_001366110.1	AGGACACGGTGAGGGTCTGGT	CAGTGGTTCCAGGGCAGGCA
USH1C	NM_153676.4	GGATGTCCGGCTCCTACGCAT	TCGATCCAGTCCCCGCCCTG
VIL1	NM_007127.2	TGGTGAAGCAGGGACACGAGC	GTGACCTCAGCAGTGATCTGGCT
