# Supplementary material for: HNF4A and HNF1A exhibit tissue specific target gene regulation in pancreatic beta cells and hepatocytes
Source: Nat Commun. 2024 Jun 22;15:4288. doi: 10.1038/s41467-024-48647-w (PMC11193738; doi:10.1038/s41467-024-48647-w)
Supplement: Supplementary file 3 — Description of Additional Supplementary Files [file 41467_2024_48647_MOESM3_ESM.pdf]

**Description of Additional Supplementary Files**

**SUPPLEMENTARY DATA LEGENDS**

**Data S1.** Peak enrichment regions identified from HNF4A ChIP-Seq across different cell types and in comparison with existing data. Related to Figures 1 and S2.

**Data S2.** Peak enrichment regions identified from HNF1A ChIP-Seq across different cell types and in comparison with existing data. Related to Figures 1 and S2.

**Data S3.** Gene ontology (GO) biological processes (BP) identified from HNF4A ChIP-Seq gene targets across multiple cell types. Analysis of GO data is based on the ChIPseeker R package (see Methods). Related to Figures 2, 6 and S3.

**Data S4.** Common HNF4A-bound beta cell gene targets identified from ChIP-Seq in D35 βLC, EndoC-βH1 cells and human islets (within 10kb upstream/downstream of TSS). Related to Figure 2.

**Data S5.** Common HNF4A-bound gene targets identified from ChIP-Seq in hepatic cells (D8 Hep and HepG2 cells). Related to Figures 4, 6 and S5.

**Data S6.** Gene ontology (GO) biological processes (BP) identified from HNF1A ChIP-Seq gene targets across multiple cell types. Analysis of GO data is based on the ChIPseeker R package (see Methods). Related to Figures 5 and 6.

**Data S7.** Common HNF1A-bound beta cell gene targets identified from ChIP-Seq in D20 EP and human islets (within 10kb upstream/downstream of TSS). Related to Figure 5.

**Data S8.** Common HNF4A- and HNF1A-bound gene targets identified from ChIP-Seq in human islets and HepG2 cells. Related to Figure 6.

**Data S9**. Components of the computed binding free energy (kcal mol−1) for HNF4A–DNA complexes. Related to Figure 7.
