# Supplementary material for: HNF4A and HNF1A exhibit tissue specific target gene regulation in pancreatic beta cells and hepatocytes
Source: Nat Commun. 2024 Jun 22;15:4288. doi: 10.1038/s41467-024-48647-w (PMC11193738; doi:10.1038/s41467-024-48647-w)
Supplement: Supplementary file 13 — Reporting Summary [file 41467_2024_48647_MOESM13_ESM.pdf]

nature portfolio

Corresponding author(s): Adrian Kee Keong Teo

Last updated by author(s): Feb 29, 2024

Reporting Summary

Nature Portfolio wishes to improve the reproducibility of the work that we publish. This form provides structure for consistency and transparency in reporting. For further information on Nature Portfolio policies, see our Editorial Policies and the Editorial Policy Checklist.

Statistics

For all statistical analyses, confirm that the following items are present in the figure legend, table legend, main text, or Methods section.

n/a	Confirmed	
☐	☒	The exact sample size (*n*) for each experimental group/condition, given as a discrete number and unit of measurement
☐	☒	A statement on whether measurements were taken from distinct samples or whether the same sample was measured repeatedly
☐	☒	The statistical test(s) used AND whether they are one- or two-sided<br>*Only common tests should be described solely by name; describe more complex techniques in the Methods section.*
☐	☒	A description of all covariates tested
☐	☒	A description of any assumptions or corrections, such as tests of normality and adjustment for multiple comparisons
☐	☒	A full description of the statistical parameters including central tendency (e.g. means) or other basic estimates (e.g. regression coefficient) AND variation (e.g. standard deviation) or associated estimates of uncertainty (e.g. confidence intervals)
☐	☒	For null hypothesis testing, the test statistic (e.g. *F*, *t*, *r*) with confidence intervals, effect sizes, degrees of freedom and *P* value noted<br>*Give P values as exact values whenever suitable.*
☒	☐	For Bayesian analysis, information on the choice of priors and Markov chain Monte Carlo settings
☒	☐	For hierarchical and complex designs, identification of the appropriate level for tests and full reporting of outcomes
☒	☐	Estimates of effect sizes (e.g. Cohen's *d*, Pearson's *r*), indicating how they were calculated

Our web collection on statistics for biologists contains articles on many of the points above.

Software and code

Policy information about availability of computer code

Data collection	AMBER 18 v18, PyMOL v1.3, ModLoop web server main.4475997, PDB2PQR v1.7
Data analysis	GraphPad Prism v9<br>FlowJo v7.0<br>BioRad CFX Manager v2.3<br>Olympus Fluoview v3.1<br>Integrative Genomics Viewer (IGV) v2.13.1<br>FASTQC<br>BBDuk<br>Bowtie2<br>Samtools<br>Sambamba<br>MACS2<br>R/Bioconductor<br>HOMER

For manuscripts utilizing custom algorithms or software that are central to the research but not yet described in published literature, software must be made available to editors and reviewers. We strongly encourage code deposition in a community repository (e.g. GitHub). See the Nature Portfolio guidelines for submitting code & software for further information.

Data

Policy information about availability of data

All manuscripts must include a data availability statement. This statement should provide the following information, where applicable:

- Accession codes, unique identifiers, or web links for publicly available datasets
- A description of any restrictions on data availability
- For clinical datasets or third party data, please ensure that the statement adheres to our policy

The ChIP-Seq dataset generated in this study have been deposited on GEO: GSE206240. The processed ChIP-Seq data generated in this study are provided as Supplementary Tables 1, 2, 3 and 6. The HepG2 ChIP-Seq data used for comparison were obtained from the ENCODE database (ENCSR000BLF and ENCSR800QIT). MD simulation input files, initial and final coordinate files have been deposited in Zenodo and are available at https://doi.org/10.5281/zenodo.10011995. The crystal structure of HNF4A used is from PDB code 4IQR. Source data are provided with this paper.

Research involving human participants, their data, or biological material

Policy information about studies with human participants or human data. See also policy information about sex, gender (identity/presentation), and sexual orientation and race, ethnicity and racism.

Reporting on sex and gender	Sex and gender are not relevant to our study as our study only utilized a donor-derived hiPSC line (iAgb) from a healthy control that was part of a previously published paper (Loo et al. 2020).
Reporting on race, ethnicity, or other socially relevant groupings	There was no human subject recruitment in our study as our study only utilized a donor-derived hiPSC line (iAgb) from a healthy control that was part of a previously published paper (Loo et al. 2020).
Population characteristics	There was no human subject recruitment in our study as our study only utilized a donor-derived hiPSC line (iAgb) from a healthy control that was part of a previously published paper (Loo et al. 2020).
Recruitment	There was no human subject recruitment in our study as our study only utilized a donor-derived hiPSC line (iAgb) from a healthy control that was part of a previously published paper (Loo et al. 2020).
Ethics oversight	There was no human subject recruitment in our study as our study only utilized a donor-derived hiPSC line (iAgb) from a healthy control that was part of a previously published paper (Loo et al. 2020).

Note that full information on the approval of the study protocol must also be provided in the manuscript.

Field-specific reporting

Please select the one below that is the best fit for your research. If you are not sure, read the appropriate sections before making your selection.

☒ Life sciences ☐ Behavioural & social sciences ☐ Ecological, evolutionary & environmental sciences

For a reference copy of the document with all sections, see nature.com/documents/nr-reporting-summary-flat.pdf

Life sciences study design

All studies must disclose on these points even when the disclosure is negative.

Sample size	No sample size calculation was conducted as this study is not a population or cohort study involving human participants. We applied the rule of at least n=3 of independent experiments or biological replicates for all in vitro experiments unless otherwise stated. As these are data obtained from clonal cell lines within controlled environments, the sample sizes are sufficient.
Data exclusions	No data excluded.
Replication	All experiments were performed at least 3 times (unless otherwise stated) and are reproducible. Number of replicates was clearly stated in the respective figure legends. For ChIP-Seq, sequencing data was not obtained from n=3 samples per antibody per cell type due to the large number of cell types present. However, ChIP validation by qPCR of prioritized gene targets were obtained from n=3 samples per cell type, and conclusions were only drawn when the results are replicated across multiple cell types or multiple antibodies used.
Randomization	Our study is a prospective biological study and does not require randomization as there are no human participants. All samples used in each set of experiments were treated equally.
Blinding	No blinding is required for this study as all cell lines were manipulated equally at the same time. Standardized automated procedures were used to acquire qPCR, ELISA and luminescence data using the respective instruments.

Reporting for specific materials, systems and methods

We require information from authors about some types of materials, experimental systems and methods used in many studies. Here, indicate whether each material, system or method listed is relevant to your study. If you are not sure if a list item applies to your research, read the appropriate section before selecting a response.

Materials & experimental systems

n/a	Involved in the study
☐	☒ Antibodies
☐	☒ Eukaryotic cell lines
☒	☐ Palaeontology and archaeology
☒	☐ Animals and other organisms
☒	☐ Clinical data
☒	☐ Dual use research of concern
☒	☐ Plants

Methods

n/a	Involved in the study
☐	☒ ChIP-seq
☐	☒ Flow cytometry
☒	☐ MRI-based neuroimaging

Antibodies

Antibodies used

Mouse monoclonal anti-HNF4A 1:1000 (WB), 1:100 (IF & Flow), 10µg (ChIP); R&D Systems H1415; RRID: AB_2263954
Mouse monoclonal anti-HNF4A 10µg (ChIP); Abcam ab41898; RRID: AB_732976
Rabbit polyclonal anti-HNF4A 10µg (ChIP); Santa Cruz sc-8987; RRID: AB_2116913
Rabbit polyclonal anti-HNF1A 1:100 (IF & Flow), 10µg (ChIP); Abcam ab96777; RRID: AB_10679303
Mouse monoclonal anti-FLAG M2 1:1000 (WB), 10µg (ChIP); Sigma Aldrich F1804; RRID: AB_262044
Mouse monoclonal anti-β-Actin 1:5000 (WB); Cell Signaling Technology 3700S; RRID: AB_2242334
Goat anti-rabbit IgG HRP 1:10000; Promega W4011; RRID: AB_430833
Goat anti-mouse IgG HRP 1:10000; Santa Cruz sc-2005; RRID: AB_631736
Donkey anti-goat IgG (H+L) Alexa Fluor 488 1:500; Thermo Fisher Scientific A11055; RRID: AB_2534102
Donkey anti-mouse IgG (H+L) Alexa Fluor 488 1:500; Thermo Fisher Scientific A21202; RRID: AB_141607
Donkey anti-rabbit IgG (H+L) Alexa Fluor 488 1:500; Thermo Fisher Scientific A21206; RRID: AB_2535792
Donkey anti-mouse IgG (H+L) Alexa Fluor 594 1:500; Thermo Fisher Scientific A21203; RRID: AB_141633

Validation

All antibodies are validated by the manufacturers.

Mouse monoclonal anti-HNF4A; R&D Systems H1415
This antibody specifically recognizes human HNF4 alpha (isoforms 1, 2, 4, 5, 7 and 8) and cross-reacts with mouse and rat HNF4 alpha (isoforms 1, 2, 4, 5, 7 and 8). It can be used in immunoblotting, immunohistochemistry, immunoprecipitation, chromatin immunoprecipitation (ChIP), direct ELISA and gel supershift assay.

Mouse monoclonal anti-HNF4A; Abcam ab41898
This is a monoclonal antibody that has been validated in flow cytometry, immunoblotting, IHC-P, ICC/IF and tested in human, rat samples.

Rabbit polyclonal anti-HNF4A; Santa Cruz sc-8987
This antibody recognizes an epitope corresponding to amino acids 295-465 mapping at the C-terminus of HNF-4α of human origin. This antibody has been discontinued and therefore our study utilizes other HNF4A antibodies.

Rabbit polyclonal anti-HNF1A; Abcam ab96777
This antibody reacts with human samples and is validated in immunoblotting, immunohistochemistry-paraffin, immunocytochemistry-immunoflourescence.

Mouse monoclonal anti-FLAG M2; Sigma Aldrich F1804
This is an affinity-purified monoclonal antibody that binds to fusion proteins containing the FLAG peptide sequence. It recognizes the FLAG peptide sequence DYKDDDDK at the N-terminus, Met-N-terminus, C-terminus, and internal sites of the fusion protein. It can be used in immunoblotting, immunoprecipitation (IP), immunohistochemisty, immunofluorescence and immunocyotchemistry. It is optimized for single banded detection of FLAG fusion proteins in mammalian expression systems. It has been validated in our study to bind specifically to FLAG-tagged constructs and no non-specific bands in an empty vector control (Supp Fig. 8).

Mouse monoclonal anti-β-Actin; Cell Signaling Technology 3700S
This is a monoclonal antibody that recognizes an epitope located on the N-terminal end of the β-isoform of actin. It specifically labels endogenous total β-actin in a wide variety of tissues and species using immunoblotting (42 kDa), immunofluorescent staining of cultured cell lines, and immunohistochemistry. It is produced against a synthetic peptide corresponding to amino-terminal residues of human β-actin. It has been validated in our study to bind to a specific band with the expected size for β-actin (Supp Fig. 8).

Eukaryotic cell lines

Policy information about cell lines and Sex and Gender in Research

Cell line source(s)

Human: H9/WA09 hESC line (NIH approval number NIHhESC-10-0062) WiCell WAe0009-A
Human: iAGb hiPSC line (Loo et al., 2020)
Human: Ad293 Agilent STR-240085
Human: HepG2 ATCC HB-8065
Human: EndoC-βH1 Univercell Biosolutions

Authentication	All commercially available cell lines were not authenticated. The hiPSC line used in this study (iAgb) was authenticated in a previous study (Loo et al., 2020) using karyotyping, confirmation of cell morphology and pluripotency staining.
Mycoplasma contamination	All cell lines were routinely tested to be negative for mycoplasma contamination.
Commonly misidentified lines (See ICLAC register)	No commonly misidentified cell lines were used.

Plants

Seed stocks	*Report on the source of all seed stocks or other plant material used. If applicable, state the seed stock centre and catalogue number. If plant specimens were collected from the field, describe the collection location, date and sampling procedures.*
Novel plant genotypes	*Describe the methods by which all novel plant genotypes were produced. This includes those generated by transgenic approaches, gene editing, chemical/radiation-based mutagenesis and hybridization. For transgenic lines, describe the transformation method, the number of independent lines analyzed and the generation upon which experiments were performed. For gene-edited lines, describe the editor used, the endogenous sequence targeted for editing, the targeting guide RNA sequence (if applicable) and how the editor was applied.*
Authentication	*Describe any authentication procedures for each seed stock used or novel genotype generated. Describe any experiments used to assess the effect of a mutation and, where applicable, how potential secondary effects (e.g. second site T-DNA insertions, mosiacism, off-target gene editing) were examined.*

ChIP-seq

Data deposition

☒ Confirm that both raw and final processed data have been deposited in a public database such as GEO.

☐ Confirm that you have deposited or provided access to graph files (e.g. BED files) for the called peaks.

Data access links *May remain private before publication.*	ChIP-Seq dataset in this study is available on GEO: GSE206240.
Files in database submission	All files available will be listed on https://www.ncbi.nlm.nih.gov/geo/query/acc.cgi?acc=GSE206240
Genome browser session (e.g. UCSC)	Not applicable.

Methodology

Replicates	ChIP-Seq data were obtained from 1 or 2 replicate samples per antibody per cell type due to the large number of cell types present. However, ChIP validation by qPCR of prioritized gene targets were obtained from 3 replicate samples per cell type, and conclusions were only drawn when the results are replicated across multiple cell types or multiple antibodies used.
Sequencing depth	The NEXTSEQ High Output was performed using the Illumina NEXTSEQ 500 Sequencers with the Illumina® Reagent v2 (75 cycle kit) Kit. The DNA were attached to the flowcell surfaces and amplified to clusters and attached with the Sequencing primers and run at 1x76cycles, generating Single-Read 75 base-pair reads.
Antibodies	Mouse monoclonal anti-HNF4A; R&D Systems H1415; RRID: AB_2263954<br>Mouse monoclonal anti-HNF4A; Abcam ab41898; RRID: AB_732976<br>Rabbit polyclonal anti-HNF4A; Santa Cruz sc-8987; RRID: AB_2116913<br>Rabbit polyclonal anti-HNF1A; Abcam ab96777; RRID: AB_10679303<br>Mouse monoclonal anti-FLAG M2; Sigma Aldrich F1804; RRID: AB_262044
Peak calling parameters	Post alignment conversion from text-based SAM files to binary BAM, sorting, and removal of duplicate and unmapped reads were done using Samtools and Sambamba. Before peak calling, the sorted BAM files were visually explored in IGV. For the peak calling step, the analysis was performed twice using MACS2, each time with a different q-value threshold (default threshold q=0.05 and relaxed threshold q=0.1).
Data quality	*Describe the methods used to ensure data quality in full detail, including how many peaks are at FDR 5% and above 5-fold enrichment.*
Software	FASTQC 0.11.9<br>BBDuk (BBMap 38.90)<br>Bowtie2 2.4.2<br>Samtools 1.11<br>Sambamba 0.8.0<br>MACS2 2.2.7<br>Bioconductor 3.12 with R 4.0.3<br>HOMER v4.11

Flow Cytometry

Plots

Confirm that:

☒ The axis labels state the marker and fluorochrome used (e.g. CD4-FITC).

☒ The axis scales are clearly visible. Include numbers along axes only for bottom left plot of group (a 'group' is an analysis of identical markers).

☒ All plots are contour plots with outliers or pseudocolor plots.

☐ A numerical value for number of cells or percentage (with statistics) is provided.

Methodology

Sample preparation	The hPSC-derived D35 cell clusters were dissociated into single cells using TrypLE at 37°C for 15min and passed through a 40µm cell strainer. Single cells were fixed with 4% paraformaldehyde for 30min, and blocked in 5% FBS in DPBS with 0.1% Triton X-100. The cells were then incubated with primary antibodies for HNF4A (H1415, R&D) or HNF1A (ab96777) at a 1:100 dilution for 1h at room temperature. Cells were washed twice and incubated with secondary antibodies for Alexa Fluor® 488 at a 1:500 dilution in the dark for 1h at room temperature. The cells were then washed twice and finally resuspended in cold DPBS for analysis.
Instrument	BD LSR II Flow Cytometer (BD Biosciences)
Software	FlowJo v7.0
Cell population abundance	No cell sorting was performed in this study.
Gating strategy	Cells were first gated on SSC-A/FSC-A to delineate the live cell population, followed by gating for single cells using FSC-H/FSC-A. Cells were stained for markers of interest using specific primary antibodies. The percentage of positively stained cells were gated against the respective secondary antibody-only control staining.

☒ Tick this box to confirm that a figure exemplifying the gating strategy is provided in the Supplementary Information.
